# Development of Clinical Skills and Confidence Questionnaire for Triage and Action Minor Emergency Course: Test-Retest Exam

**DOI:** 10.7759/cureus.17864

**Published:** 2021-09-10

**Authors:** Kenji Numata, Tomoyasu Matsubara, Yoshiki Okumura, Keita Kondo, Mano Shirakami, Daiki Kobayashi

**Affiliations:** 1 Emergency Department, St. Marianna University Hospital, Kawasaki, JPN; 2 Neurology, Hiroshima University Graduate School of Biomedical and Health Sciences, Hiroshima, JPN; 3 Emergency Medicine, Fukuchiyama Hospital, Fukuchiyama, JPN; 4 Internal Medicine, Fujita Health University, Nagoya, JPN; 5 Emergency Medicine, Otowa Hospital, Kyoto, JPN; 6 Internal Medicine, Seiroka Kokusai Byouin, Tsukiji, JPN

**Keywords:** foreign bodies in ear or nose, eye injury, ankle sprain, nose bleed, burn first aid, questionnaire development and validation, • simulation in medical education, emergency medicine

## Abstract

Aim: The Triage and Action (T&A) minor emergency course was developed to improve the clinical skills of Japanese non-specialist physicians for minor emergent problems. Currently, the course quality is evaluated only by a self-reported satisfaction questionnaire. This study described a new clinical skills and confidence questionnaire to evaluate its validity and reliability.

Methods: The web-based questionnaire was evaluated by 103 physicians identified from a mailing list as having taken the T&A minor emergency course. The clinical experience and confidence (CEC) questionnaire was prepared, and its content and contextual validity were validated using a clinical sensibility test (CST). Reliability was assessed by the interclass correlation coefficient after two weeks via a follow-up CEC questionnaire.

Results: Of the 103 physicians contacted 44 (42.7%) responded to the questionnaire, 36 (40.8%) to the follow-up CEC questionnaire, and 33 (32.0%) to both questionnaires; 28 (27.2%) participants took the clinical sensibility test. Five questions which asked the total number of patients treated within six months showed fair agreement on the reliability test. All answers to the questions in the CST were favorable.

Conclusion: We removed every question which asked the total number of patients treated for various minor emergencies within six months from CEC. Consequently, the new questionnaire was shown to be contextually well validated and reliable. We will use the CEC questionnaire to improve our course, which we hope to demonstrate improved primary care for selected minor conditions.

## Introduction

The practice of emergency medicine in Japan is different from that in western countries [1]. Adequate emergency physicians are unavailable, and non-emergency physicians are required to manage patients with minor emergent problems in the emergency room. These deficits have led to ambulances not being able to find hospitals with appropriate resources to treat patients. A frequent reason given for refusal is “without a specialist,” in case of minor emergencies [2].

We developed a Triage and Action (T&A) minor emergency course (http://minoremergency.club/) in Japan and began offering it in 2015 with simulation training to improve clinical knowledge and skills; simulation training has been reported to have clinical context validity in various studies [3-5]. The course has been held 21 times, and 461 physicians have completed it as of December 31, 2018. The course aims to improve clinical knowledge and skills for managing minor emergencies. The training sessions comprised five minor emergencies (epistaxis, ear and nose foreign body, sprain or fracture, ocular surface foreign bodies, and burns) with a lecture- and simulation-based training conducted by dedicated multidisciplinary instructors. The simulation-based training uses real-case scenarios, and participants decide how to treat each training-simulated patient with a minor emergency. The instructors described patients with various diseases and injury scenarios and assessed the participants’ decisions and skills. This is a one-day course (7 h), and two T&A minor emergency instructors supervise five attendants. Instructors for the T&A minor emergency course are selected by a T&A principal member after taking the course. All 441 participants who answered the post-course-paper-based “satisfaction” questionnaire responded positively when asked, “Do you think you can use lessons learned from this course in practice?” 80.1% responded “Strongly agree” and 19.9% responded “Agree” [responses from the Likert scale (strongly agree, agree, neither agree nor disagree, disagree, and strongly disagree)]. However, information regarding the physicians’ clinical practice in treating minor emergencies after taking the course has been limited.

Improvements in clinical skills have usually been evaluated by performing the same simulation task or in a real clinical situation more than three weeks after the simulation [6]. However, T&A minor emergency course participants from all over Japan have participated in this course, making it difficult to evaluate the change in a particular physician’s skill level. Therefore, we considered that information regarding a physician’s confidence level and the total number of minor emergencies treated could be used to show the change in the physicians’ practice and reduce their refusals to treat minor emergencies.

Some simulation courses have used questionnaires to evaluate knowledge before and after the simulation course [7-9]. To ensure that the questionnaire closely matches the real-world experience and ability, the post-course questionnaire should be completed within a few months and should contain information about self-confidence and real clinical practice experience (e.g., change in the total number of treatments without referrals to a specialist within the specific period).

A questionnaire was used to evaluate the change of confidence or clinical skills among physicians [10]. However, to our knowledge, articles on the validity and reliability of questionnaires and changes in physicians’ confidence and attitude before and after few months of taking the course are limited. Herein, we aimed to develop a clinical experience and confidence (CEC) questionnaire to evaluate its validity and reliability.

## Materials and methods

This study was approved by the Ethics Committee of the Tokyo Bay Urayasu/Ichikawa Hospital (approval number: 385).

Evaluation strategy

We conducted this study in accordance with the method proposed by Burns et al. [11]. The survey used a questionnaire and the clinical sensibility test (CST). We had previously developed the questionnaire to evaluate how participants change after taking a T&A minor emergency course. We tested the reliability of the questionnaire and used a CST to validate its content.

Methods

The participants responded to the first CEC questionnaire to validate its content using a CST, followed by responding to the follow-up CEC questionnaire within two weeks after the test to evaluate reliability (test-retest exam). This survey was conducted in November and December 2018.

Participants

The web-based questionnaire was evaluated by 103 physicians identified from a mailing list as having taken the T&A minor emergency course. There were two inclusion criteria. (1) To evaluate reliability, we included the responders who answered the first and follow-up CEC questionnaires. (2) To evaluate the validity, we included physicians who answered the first questionnaire and took the CST.

Questionnaire

Two principal developers of the T&A minor emergency course and one physician-researcher created a new questionnaire, called the CEC questionnaire. The questionnaire comprised 32 questions related to two major factors: the physicians’ background and practice experience and self-confidence in treating minor emergencies (Supporting Information 1). The responses were provided by checking boxes or by ranking responses.

In the first section, the data collected included age, sex, post-graduate year, specialty (residents, 19 basic specialists from the Japanese Society of Internal Medicine, and others), and the number of hospital beds (clinic and 20-49, 50-99, 100-199, 200-299 beds, 300-499, and ≥500 beds) [12].

The second section of the CEC questionnaire had questions about the physicians’ experience and confidence in treating each type of minor emergency. We chose five minor emergencies (epistaxis, ear and nose foreign body, sprain or fracture, burn, and ocular surface foreign bodies) that were presented via simulation-based training in the T&A minor emergency course. To evaluate the participant’s hospital for its ability to provide minor emergency treatment, it was required that the hospital has a specialist for the disease; for example, the specialist for epistaxis and ear and nose foreign body was otolaryngologists [13,14], for sprain or fracture were orthopedic physicians [15], for burns were dermatologists and plastic surgeons [16], and for ocular surface foreign bodies were ophthalmologists [17]. The following questions were created: (1) “The total number of patients seen within each specialty in a month,” (2) “With or without specialists in the respondent’s hospital,” (3) “Total number of minor emergencies treated within six months,” (4) “Confidence,” (5) Experience of each minor emergency without a specialist’s support within the last six months?,” and (6) “Total number of patients treated for each minor emergency without specialist support within the last six months?”

The web-based follow-up CEC questionnaire was modified and resent to participants two weeks after completion of the first CEC questionnaire. The modification was the removal of the physician’s background in the first questionnaire to reduce the effort needed to answer the follow-up CEC questionnaire.

To evaluate the reproducibility of the questionnaire, the participant’s name was used to match the first and follow-up CEC questionnaires’ responses.

Clinical sensibility test (CST)

The CST was performed to assess the comprehensiveness, clarity, and contextual validity of the web-based CEC questionnaire’s content (Supporting Information 2) [11]: (1) “Important issues pertaining to the T&A course,” (2) “Missing items,” (3) “Simplicity and ease in understanding,” (4) “Information about physician’s knowledge and experience,” (5) Inappropriate or redundant items,” (6) “Issues in the physician’s knowledge and experiences of minor emergencies,” and (7) “Answering time (minutes).” The participants answered questions 1-4 and 6 by selecting a response from the Likert scale (e.g., very unlikely, unlikely, neutral, likely, and very likely). The answers to question 5 were “Yes” or “No.” Questions 2, 4, 5, and 6 had a free-entry column about each question. A free-entry column was also provided for the participants to suggest ideas on how to improve the questionnaire.

Primary data analysis

We used the STATA/MP 15.1 software (StataCorp LLC, Texas, USA) for data analyses and interclass correlation coefficient (ICC) analysis to assess reliability. We decided that the reliability coefficient could be qualitatively categorized as follows: ICC < 0.4 is poor, 0.4 ≤ ICC < 0.6 is fair, 0.6 ≤ ICC < 0.75 is good, and 0.75 ≤ ICC ≤ 1 is excellent [18]. 

## Results

Characteristics of study participants

During the study period, 44 (42.7%) participants responded to the first CEC questionnaire and 36 (40.8%) responded to the follow-up CEC questionnaire; 33 (32.0%) answered both questionnaires; 28 (27.2%) took the CSC, and no responders replied only to the CSC. Table 1 presents the backgrounds of the physicians who answered both CEC questionnaires.

**Table 1:** Characteristics of the participants who took the CEC and follow-up CEC questionnaires IQR - Interquartile range; CEC - Clinical experience and confidence; PGY - Post-graduate year

Variable	First questionnaire (n = 33)
Age, years, median (IQR)	34 (31–39)
PGY, years, median (IQR)	8.0 (5–10)
Male gender, No. (%)	31 (93.9)
Specialty, No. (%)	
Residents	3 (9.1)
Internal medicine	8 (24.2)
Surgery	0 (0.0)
General medicine	8 (24.2)
Pediatric	1 (3.0)
Plastic surgery	0 (0.0)
Emergency medicine	6 (18.2)
Pathology	0 (0.0)
Anesthesiology	0 (0.0)
Radiology	0 (0.0)
Neurosurgery	0 (0.0)
Urology	0 (0.0)
Otolaryngology	0 (0.0)
Ophthalmology	1 (3.0)
Gynecology	0 (0.0)
Orthopedic	5 (15.2)
Psychiatry	0 (0.0)
Dermatology	1 (3.0)
Rehabilitation	0 (0.0)
Clinical laboratory department	0 (0.0)
Others	0 (0.0)
Size of hospital, No. (%)	
Clinic	4 (12.5)
20–49 beds	1 (3.1)
50–99 beds	2 (6.3)
100–199 beds	6 (18.8)
200–299 beds	1 (3.1)
300–499 beds	8 (25.0)
≥500 beds	10 (31.3)

The first CEC and the follow-up CEC

Table 2 presents the results of the participants who took the first CEC and the follow-up CEC questionnaires. The mean time period in which the two questionnaires were completed was 35.4 days (SD = 12.1). We found that five questions regarding “The total number of epistaxis patients treated without an otolaryngologist within 6 months”; “The total number of ear and nose foreign body patients treated without an otolaryngologist within 6 months”; “The total number of burn patients treated within 6 months”; “The total number of burn patients treated without a dermatologist or plastic surgeon within 6 months”; and “The total number of patients with ocular surface foreign bodies treated without an ophthalmologist within 6 months” achieved a fair ICC (0.4 ≤ ICC < 0.6).

**Table 2:** Results of ICC in the first CEC and follow-up CEC questionnaires IQR - Interquartile range; ICC - Interclass correlation coefficient;  CEC - Clinical experience and confidence

Variables	First questionnaire (n = 33)	Follow-up questionnaire	ICC
Otolaryngologists			
Epistaxis			
1. Otolaryngologist, No. (%)	17 (51.5)	16 (48.5)	0.97
2. Total number of patients with otolaryngology disease treated within 1 month, median (IQR^a^)	2.0 (1.0–5.0)	2.0 (1.0–5.0)	0.61
3. Total number of epistaxis patients treated within 6 month, median (IQR)	2.0 (1.0–3.0)	1.0 (0.0–2.0)	0.85
4. Confidence for “Epistaxis,” median (IQR)	3.0 (3.0–4.0)	4.0 (3.0–4.0)	0.84
5. Treated “Epistaxis” without otolaryngologist within 6 months, No. (%)	21 (65.6)	18 (54.6)	0.86
6. Total number of “Epistaxis” patients treated without otolaryngologist within 6 months, median (IQR)	1.0 (0.0–3.0)	1.0 (0.0–3.0)	0.51
Ear and nose foreign body			
3. Total number of “ear and nose foreign body” patients treated within 6 months, median (IQR)	0.0 (0.0–1.0)	0.0 (0.0–1.0)	0.76
4. Confidence for “ear and nose foreign body,” median (IQR)	3.0 (2.0–3.0)	3.0 (2.0–3.0)	0.85
5. Treated “ear and nose foreign body”without otolaryngologist within 6 months, No. (%)	14 (42.4)	8 (24.2)	0.65
6. Total number of patients with “ear and nose foreign body” treated without an otolaryngologist within 6 months, median (IQR)	0.0 (0.0–1.0)	0.0 (0.0–1.0)	0.59
Orthopedic			
Sprain or fracture			
1. Orthopedist, No. (%)	26 (78.9)	25 (75.8)	0.96
2. Total number of patients with orthopedic disease treated within a month, median (IQR)	10.0 (4.0–50.0)	10.0 (4.5–25.0)	0.99
3. Total number of patients with “sprain or fracture” treated within 6 month, median (IQR)	10.0 (2.0–50.0)	5.0 (2.0–50.0)	0.99
4. Confidence for “sprain or fracture,” median (IQR)	3.0 (3.0 –4.0)	4.0 (3.0–4.0)	0.94
5. Treated “sprain or fracture” without orthopedist within 6 months, No. (%)	17 (51.5)	17 (51.5)	0.93
6. Total number of patients with “sprain or fracture” treated without an orthopedist within 6 months, median (IQR)	2.5 (0.0–27.5)	3 (0.0–20.0)	0.69
Dermatology or plastic surgery			
Burn			
1. Dermatologist or plastic surgeon, No. (%)	20 (60.6)	20 (60.6)	0.93
2. Total number of patients with dermatological disease treated within a month, median (IQR)	8.0 (3.0–10.0)	5 (4.0–10.0)	0.61
3. Total number of “burns” patients treated within 6 months, median (IQR)	1.0 (0.0–3.0)	1.0 (0.0–3.0)	0.49
4. Confidence for “burns,” median (IQR)	3.0 (3.0–4.0)	3.0 (3.0–4.0)	0.92
5. Treated “burns” without a dermatologist or plastic surgeon within 6 months, No. (%)	19 (57.6)	16 (48.5)	0.83
6. Total number of patients with “burns” treated without a dermatologist or plastic surgeon within 6 months, median (IQR)	1.0 (0.0–2.5)	0.5 (0.0–3.0)	0.42
Ophthalmology			
Ocular surface foreign body			
1. Ophthalmologist, No. (%)	19 (57.6)	19 (57.6)	0.94
2. Total number of patients with ophthalmic disease treated within a month, median (IQR)	1.0 (1.0–5.0)	2.0 (0.0–5.0)	0.98
3. Total number of patients with “cornea and conjunctival foreign body” treated within a month, median (IQR)	1.0 (0.0–2.0)	1.0 (0.0–2.0)	0.64
4. Confidence for “ocular surface foreign body,” median (IQR)	3.0 (2.0–4.0)	3.0 (3.0–4.0)	0.86
5. Treated “ocular surface foreign body” without an ophthalmologist within 6 months, No. (%)	15 (45.5)	11 (33.3)	0.90
6. Total number of patients with “ocular surface foreign body” treated without ophthalmologist within 6 months, median (IQR)	0.0 (0.0–2.0)	0.0 (0.0–1.5)	0.48

Clinical sensibility test (CST)

Table 3 presents the results of the CST. For questions 1, 3, and 4, >80% of the respondents answered: “Fair to a large extent.” For question 2, 96.5% of the respondents opted for “Minor to insignificant gaps.” For question 5, 92.9% of the respondents stated that no items were inappropriate or redundant in the questionnaire. For question 6, 92.6% of the respondents answered “Likely to very likely.” The median time to answer the questionnaire was 5 minutes [interquartile range (IQR): 5-10 minutes].

**Table 3:** Results of participants who took the CST CST - Clinical sensibility test

Variables	Answer (n = 22)
1. To what extent are the questions directed at important issues pertaining to T&A minor emergency course participants?	
Small extent	0 (0%)
Limited extent	2 (7.1%)
Fair extent	12 (42.9%)
Moderate extent	11 (39.3%)
Large extent	3 (10.8%)
2. Are there important issues pertaining to your practice that should be included in the questionnaire but were omitted?	
Crucial gaps	0 (0%)
Important gaps	1 (3.6%)
Minor gaps	4 (14.3%)
Minimal gaps	15 (53.6%)
Insignificant gaps	8 (28.6%)
3. To what extent are the response options provided simple and easily understood?	
Small extent	0 (0%)
Limited extent	5 (17.9%)
Fair extent	12 (42.9%)
Moderate extent	7 (25.0%)
Large extent	4 (14.3%)
4. To what extent are questions likely to elicit information pertaining to your use of and experience in T&A minor emergency course’s participants?	
Small extent	0 (0%)
Limited extent	2 (7.1%)
Fair extent	10 (35.7%)
Moderate extent	10 (35.7%)
Large extent	6 (21.4%)
5. Are there any items inappropriate or redundant?	
Yes	2 (7.1%)
No	26 (92.9%)
6. How likely is the questionnaire to elicit important issues in physician’s attitude for minor emergencies in T&A minor emergency course’s participants?	
Small extent	0 (0%)
Limited extent	2 (7.1%)
Fair extent	13 (46.4%)
Moderate extent	5 (17.9%)
Large extent	8 (28.6%)
7. How long did it take you to complete the questionnaire (min)?	5 (5–10)

## Discussion

The study results showed that the CEC questionnaire about the T&A minor emergency course was valid and reliable.

In the CEC and follow-up CEC questionnaire analysis, five questions about the total number of patients treated for various minor emergencies alone or with a specialist within six months gave a fair ICC value (0.4 ≤ ICC < 0.6). Morita et al. reported that it was difficult to show reliable results for questions involving human emotions or knowledge because of changes in the subjects’ subjective conditions [19]. This fair ICC may have been caused by random error or because the participants saw different number of patients during the study periods. The results showed that total number of patients treated for various minor emergencies within six months was small and some participants did not experience them at all. Therefore, we considered questions asking for the total number of patients treated for various minor emergencies within six months were not informative enough, and the binominal questions which asked whether the participants experienced treating minor emergencies alone within six months were sufficient. Finally, we removed every question which asked the total number of patients treated for various minor emergencies alone or with a specialist within six months.

All answers to the questions in the CST were favorable, which we interpreted as strong indication of the validity of the questionnaire’s content and clinical context [20]. Regarding question 2, some responders mentioned that this survey did not include a question about “satisfaction rate.” The lack of this question may cause less confidence in the questionnaire among some physicians. However, our course already collected information regarding the “satisfaction rate” on paper-based questionnaire without the physicians’ names before starting the study. Because we thought that the participants’ answers may be affected if the questionnaire was not anonymous, no name should be associated with the “satisfaction rate” question [21]. Therefore, a question about the “satisfaction rate” was not included in our web-based questionnaire. Galesic et al. reported in their web-based study that their online questionnaire’s length (10, 20, and 30 min) was longer than the length and number of participants in our study and fewer respondents started and completed the questionnaire [22]. Our study showed that the median time to answer the questionnaire was 5 (IQR, 5-10) min, which we considered to be very reasonable.

One of the participants commented in the free-entry column that “This questionnaire should contain questions about the availability of a specialist at night or when the primary physician has a day off.” Therefore, we added a question asking “Do you have specialist support at night or on holidays?,” and the answers were “Any time as needed,” “Sometimes,” and “Not at all.”

Limitations

This study has several limitations. The first concerns the external validity because we chose T&A minor emergency mailing list members to serve as participants; therefore, the risk of selection bias should be considered. Furthermore, the limited sample size should be considered. The second is that we used two questionnaires over a two-week period. Therefore, the actual practice pattern may have changed during the two weeks. Third, the sample size was small, and the response rate was low, which can be attributed to the fact that the respondents were volunteers.

## Conclusions

This newly developed questionnaire was developed to evaluate the change in clinical skills and confidence of clinical practice after completing a T&A minor emergency course. In the future, we plan to send a pre-web-based questionnaire before the course and a post-web-based questionnaire six months after the course. The post-web-based questionnaire contains the same question as those in the pre-questionnaire, except the addition of the question “Did you change your place of work after answering the pre-questionnaire?” to check this possibility. Our study shows the validity and reliability of the questionnaire. Future research should focus on administering the questionnaire to participants in the T&A minor emergency course. These results will provide us information regarding ways to improve the T&A minor emergency course.

## Figures and Tables

**Figure 1:**
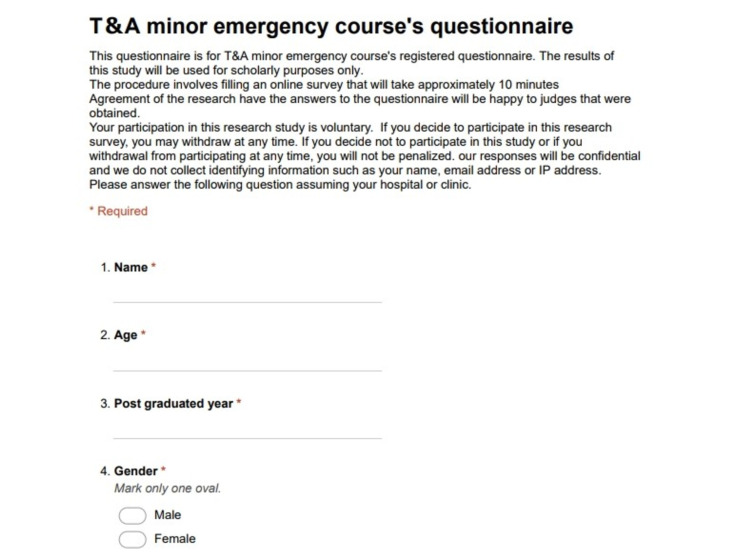
Supplement1 Page 1

**Figure 2:**
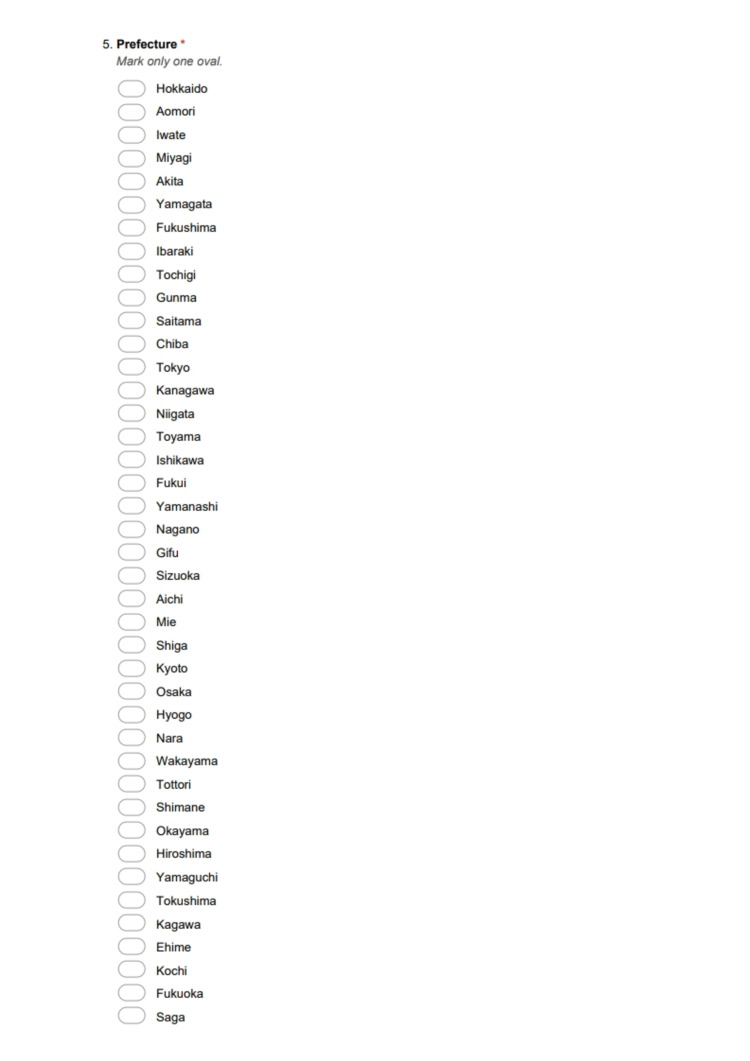
Supplement1 Page 2

**Figure 3:**
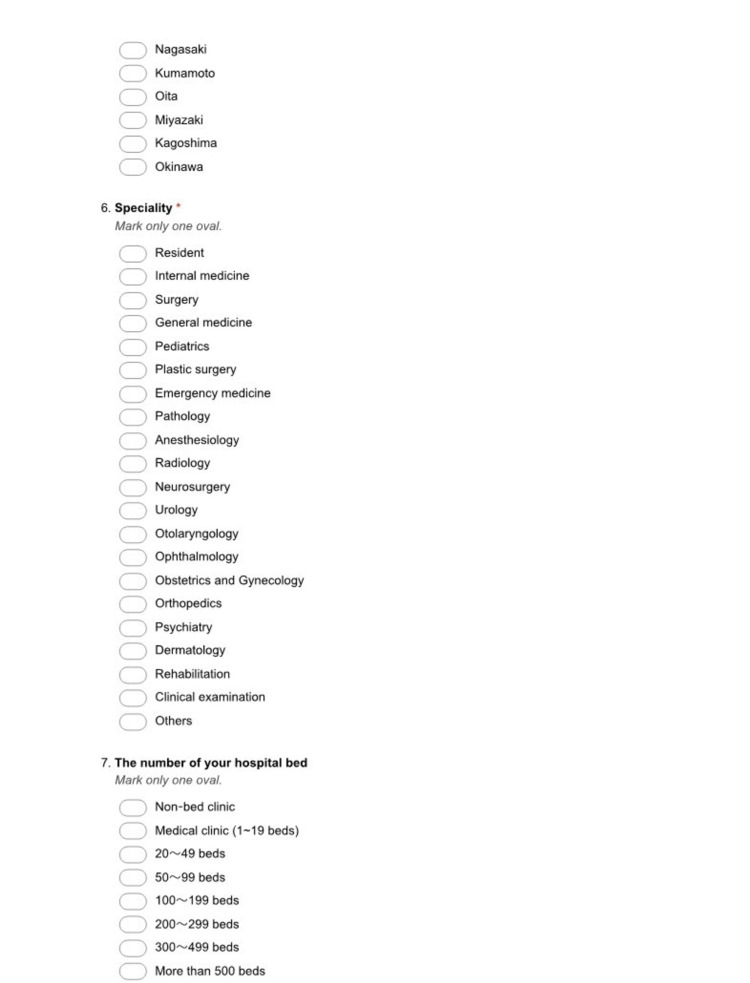
Supplement1 Page 3

**Figure 4:**
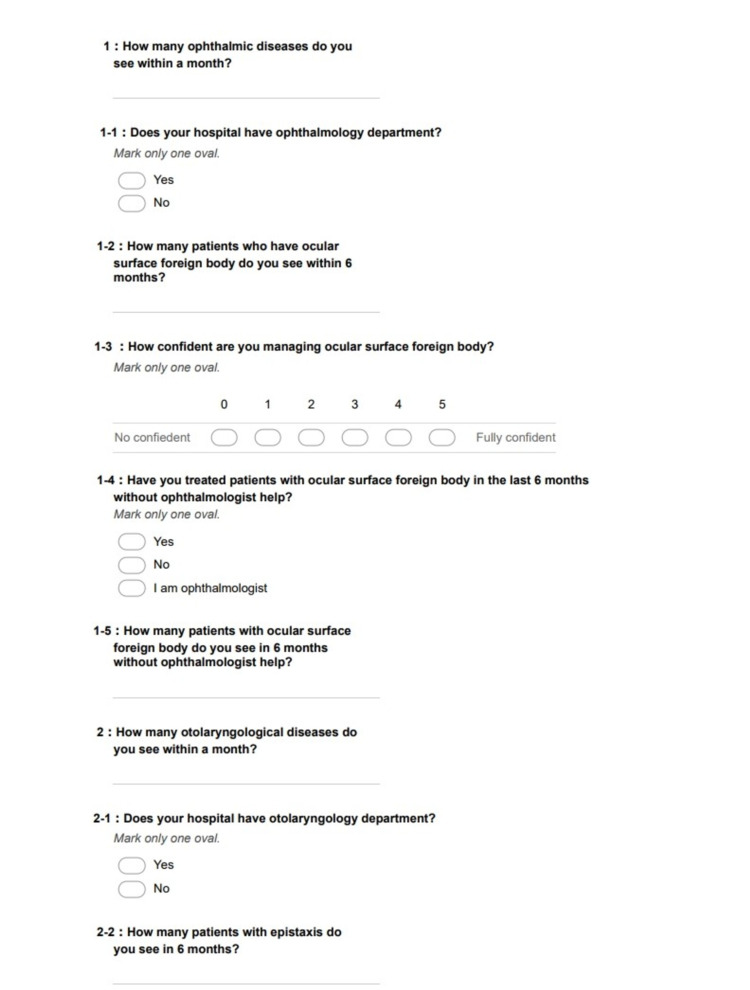
Supplement1 Page 4

**Figure 5:**
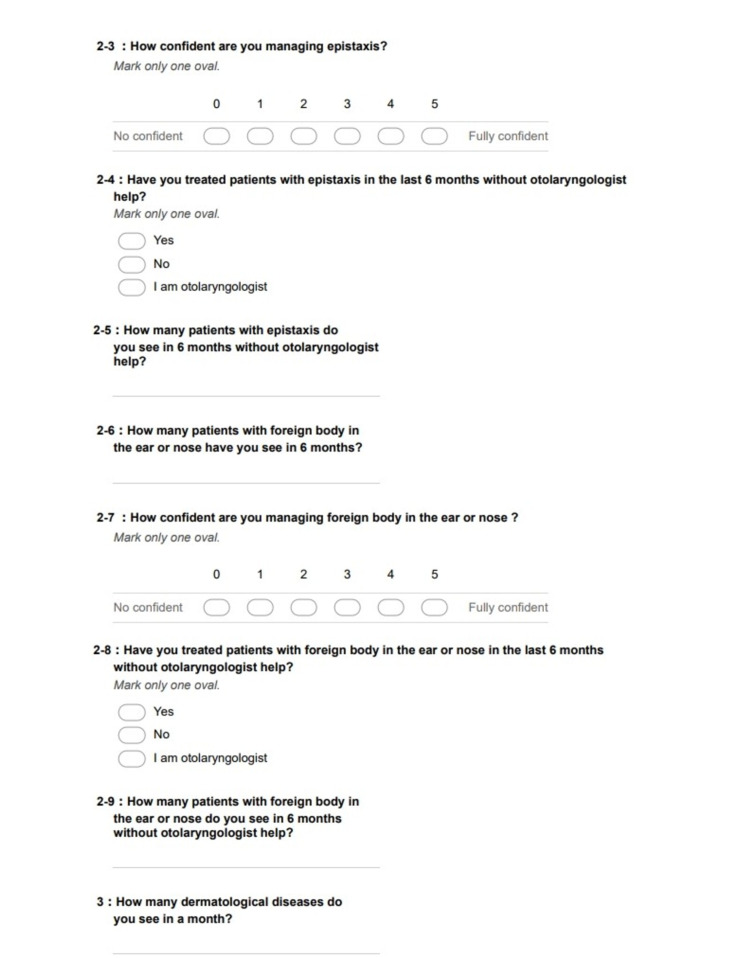
Supplement1 Page 5

**Figure 6:**
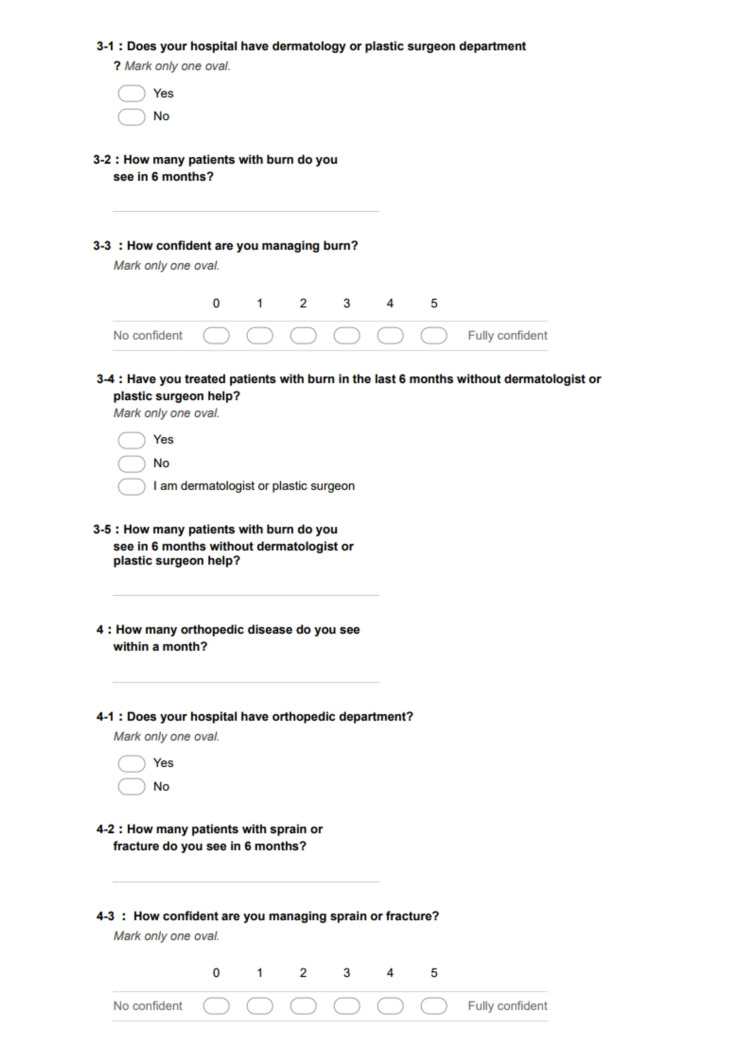
Supplement1 Page 6

**Figure 7:**
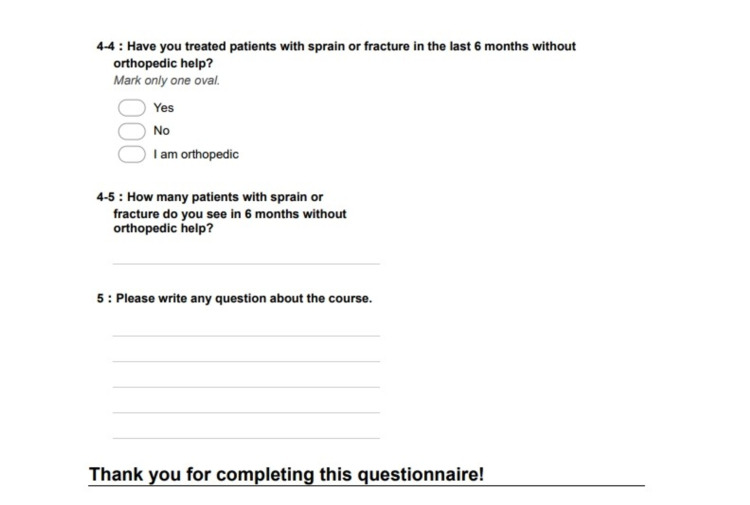
Supplement1 Page 7

**Figure 8:**
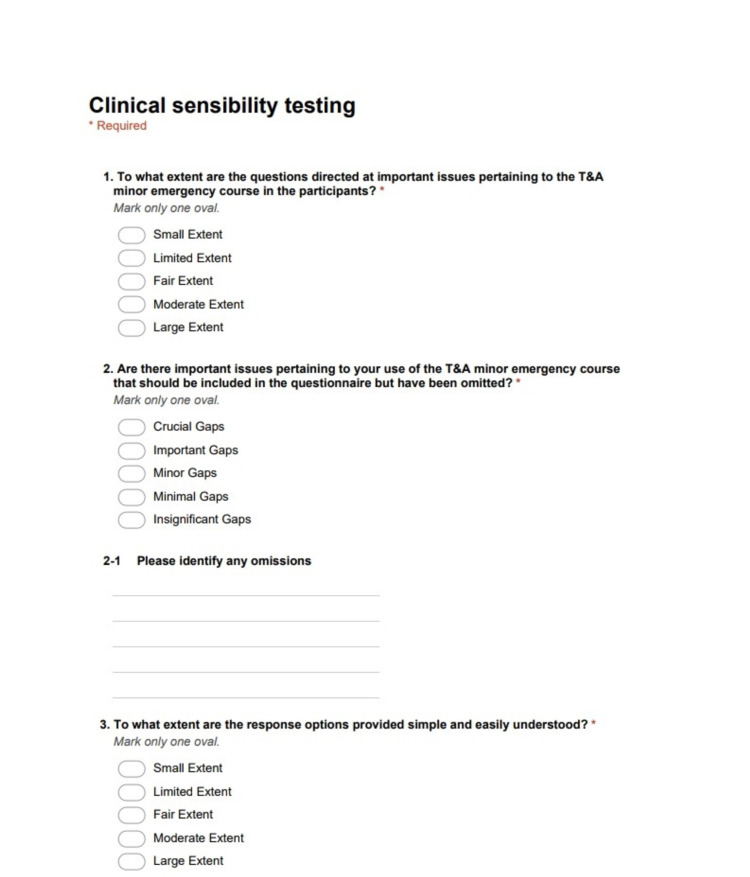
Supplement2 Page 1

**Figure 9:**
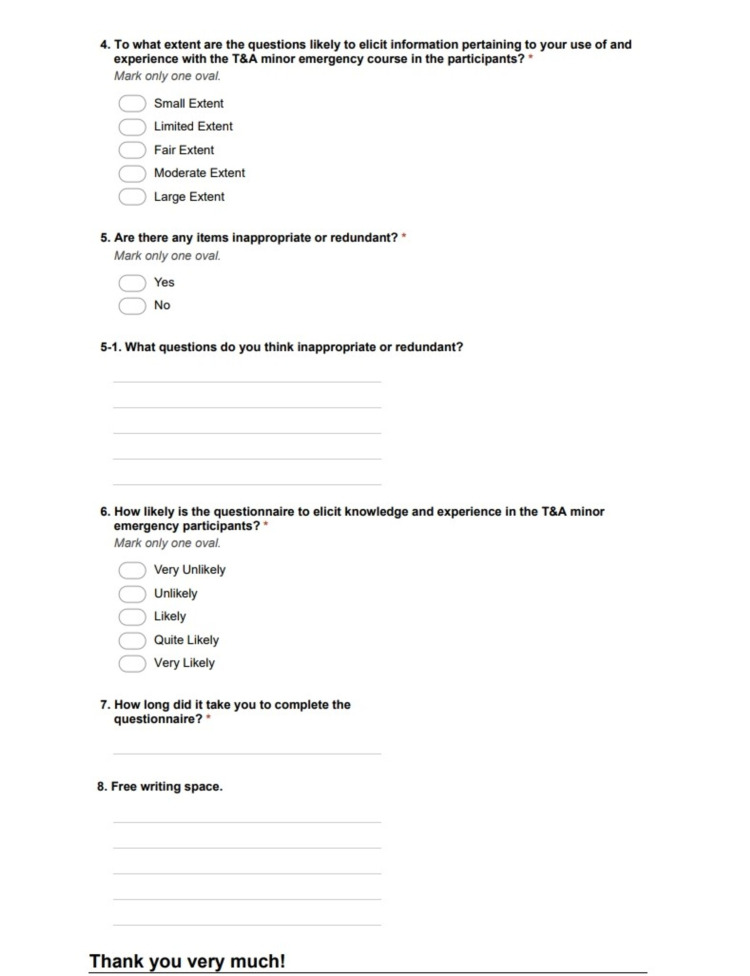
Supplement2 Page 2

**Figure 10:**
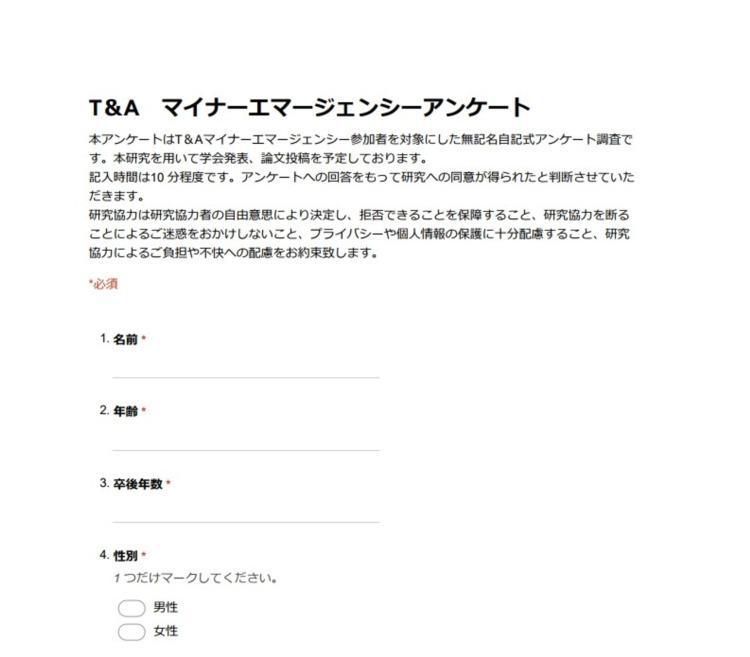
Original Japanese questionnaire page 1

**Figure 11:**
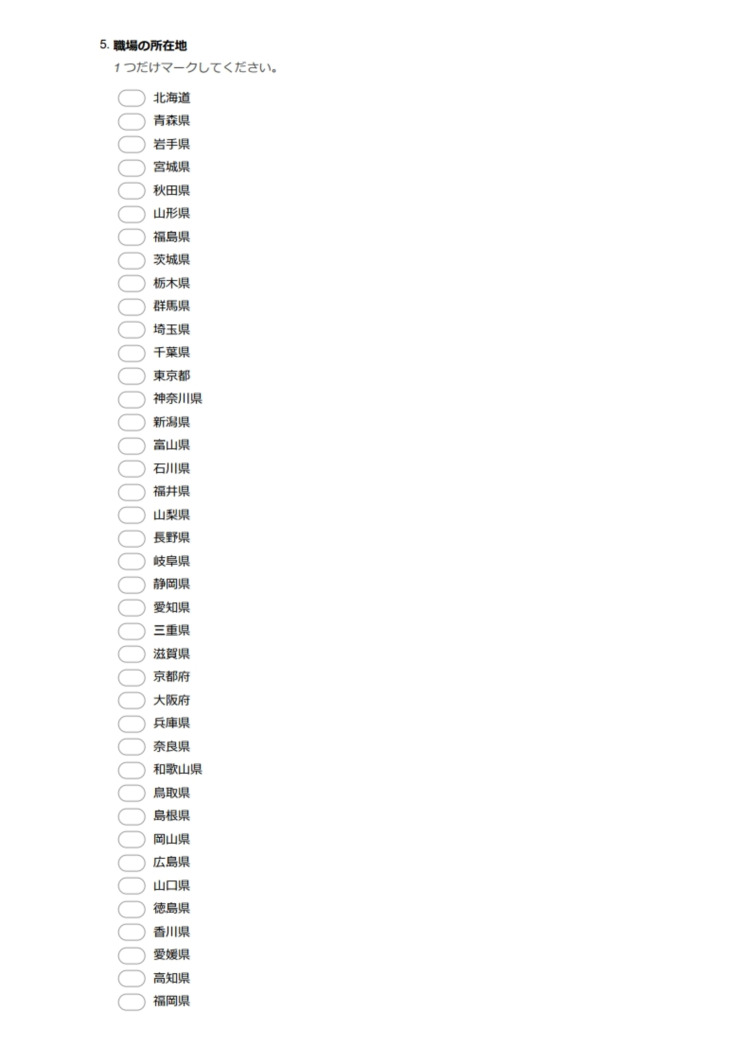
Original Japanese questionnaire page 2

**Figure 12:**
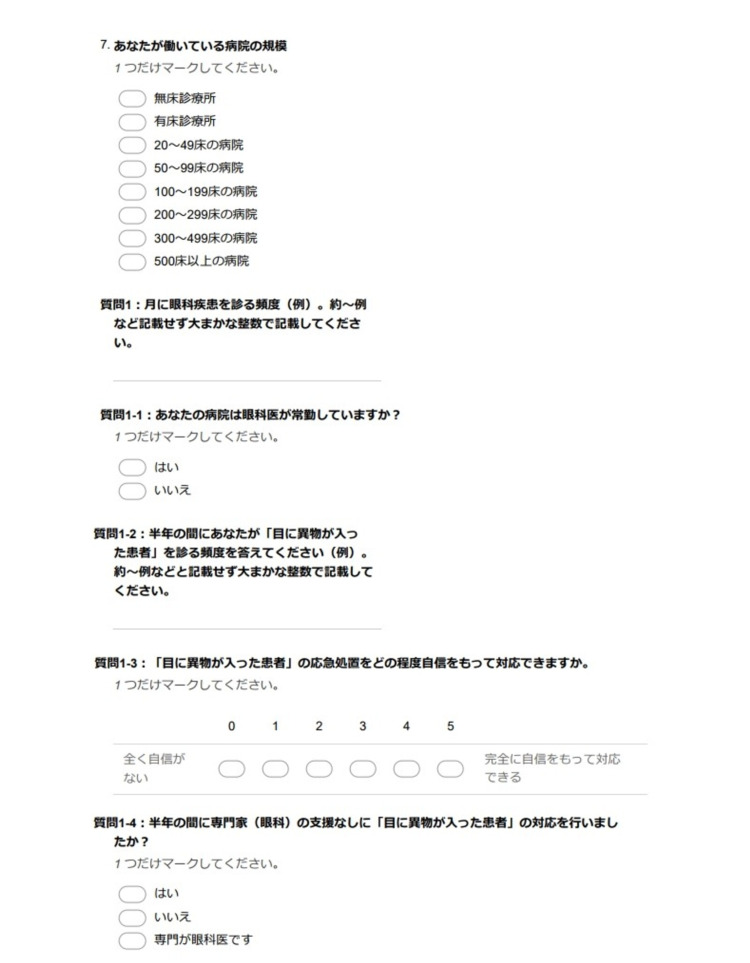
Original Japanese questionnaire page 3

**Figure 13:**
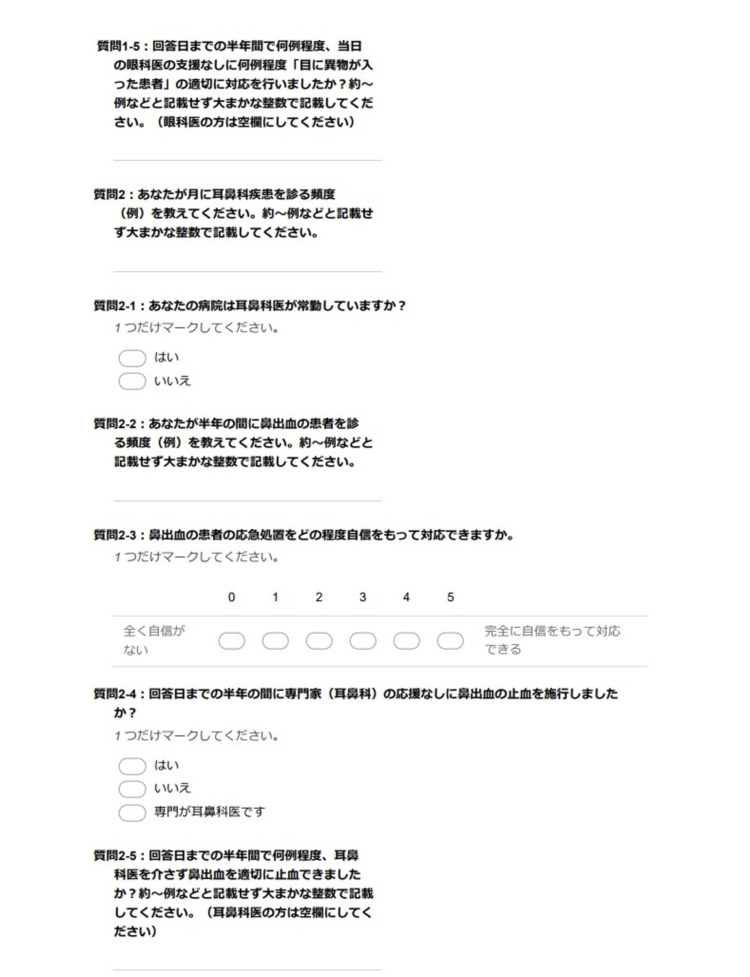
Original Japanese questionnaire page 4

**Figure 14:**
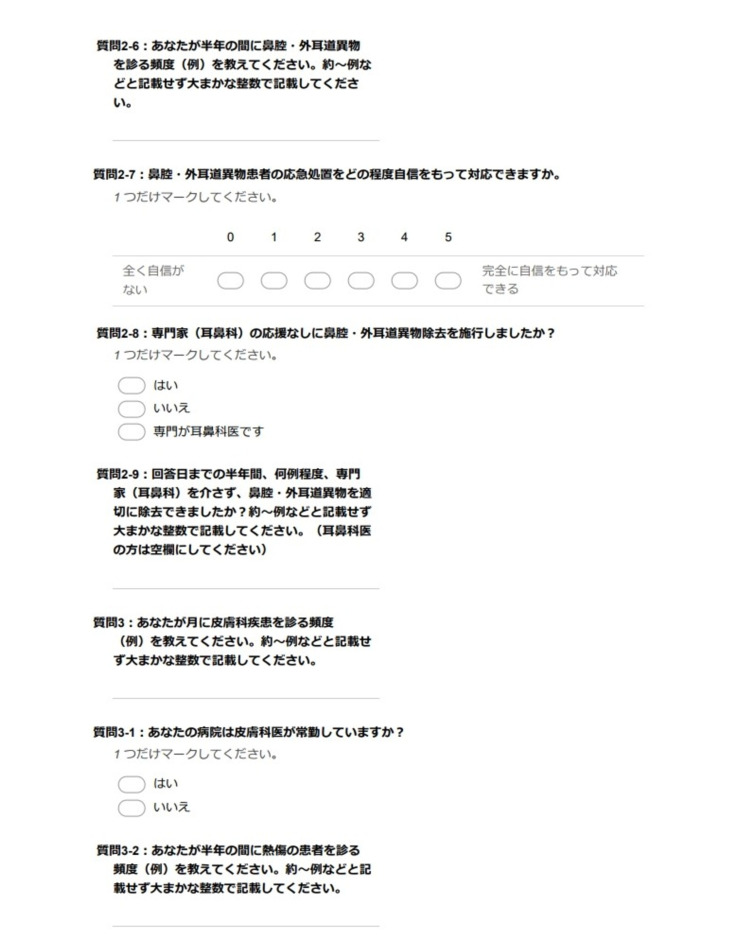
Original Japanese questionnaire page 5

**Figure 15:**
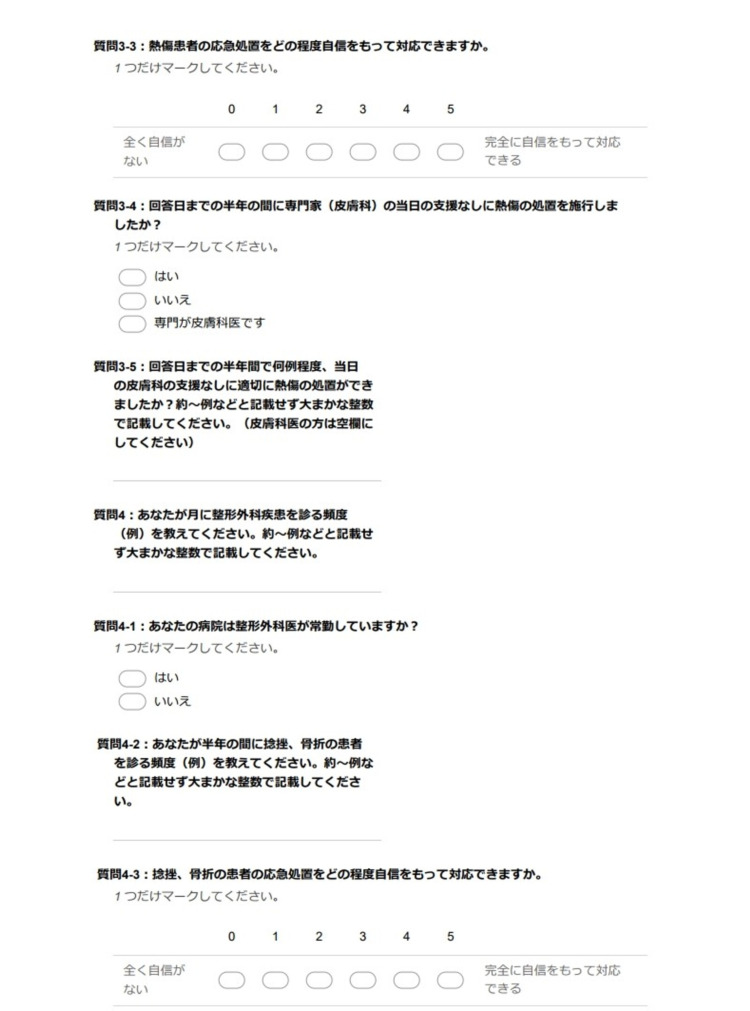
Original Japanese questionnaire page 6

**Figure 16:**
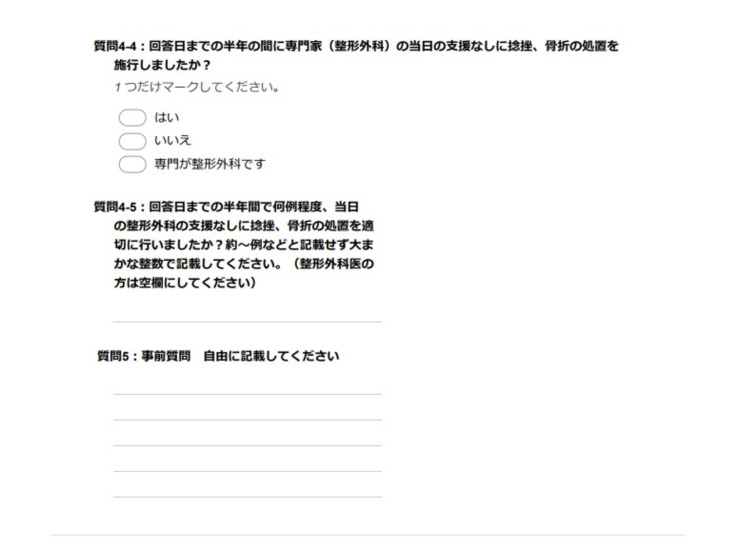
Original Japanese questionnaire page 7

**Figure 17:**
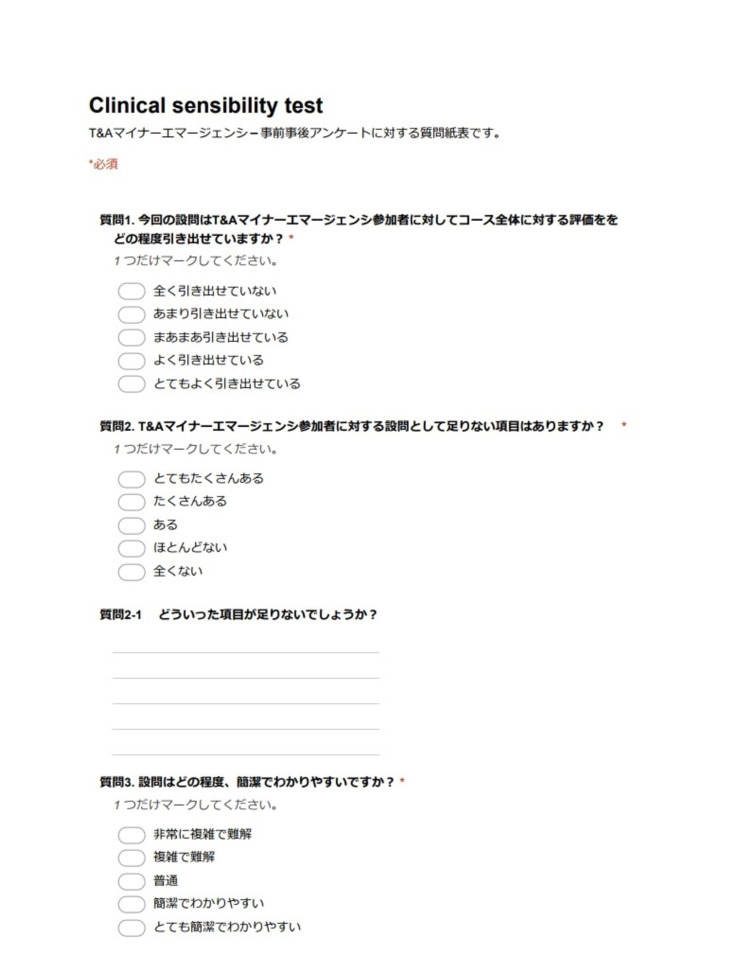
Original Japanese Clinical sensibility test page 1

**Figure 18:**
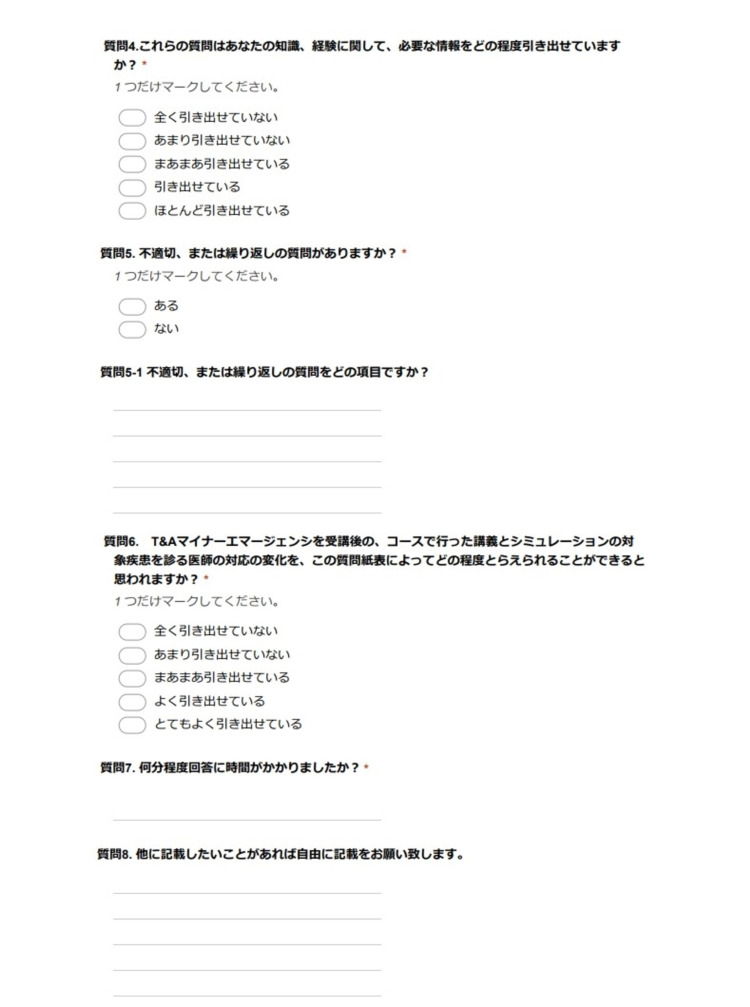
Original Japanese Clinical sensibility test page 2
